# Antitumor Effects of Chemlali and Wild Olive Tree Extracts: Role in Cell Proliferation and Apoptosis in Prostate (PC3) and Breast (MDA‐MB‐231) Cancer Cell Lines

**DOI:** 10.1155/bmri/4911257

**Published:** 2025-12-11

**Authors:** Radhia Bouazizi, Lamjed Bouslama, Fatma Nouira, Alice Grossi, Mattia De Vita, Bechir Baccouri, Selim Jallouli, Samantha Solito, Monica Savio, Virginie Sottile, Leila Abaza

**Affiliations:** ^1^ Laboratory of Biotechnology of Olive, Centre of Biotechnology of Borj Cedria, Hammam-Lif, Tunisia; ^2^ Faculty of Mathematical, Physical and Natural Sciences of Tunis, University of Tunis El Manar, Tunisia, utm.rnu.tn; ^3^ Laboratory of Bioactive Substances, Centre of Biotechnology of Borj Cedria, Hammam-Lif, Tunisia; ^4^ Department of Molecular Medicine, Immunology and General Pathology Unit, University of Pavia, Pavia, Italy, unipv.eu; ^5^ Centro Grandi Strumenti (CGS), University of Pavia, Pavia, Italy, unipv.eu; ^6^ UOC Bioscaffolds and Transplantation, Fondazione IRCCS Policlinico San Matteo, Pavia, Italy, sanmatteo.org

**Keywords:** antitumoral activity, apoptosis, breast cancer (MDA-MB-231), olive leaf, olive pomace, prostate cancer (PC3)

## Abstract

Cancer remains a major global health challenge, requiring natural therapeutic solutions. Olive tree by‐products, like leaves and pomace, are rich in bioactive phenolics with antioxidant and anticancer potential. This study explores the chemical composition and anticancer potential of the hexane fraction of olive leaf extracts and the dichloromethane fraction of olive pomace extracts from two *Olea europaea* varieties: e cultivated Chemlali (var. *europaea*) and the wild olive (*var. sylvestris*). GC‐MS analysis identified stigmast‐5‐en‐3‐ol, erythrodiol, and phytol as the predominant compounds in wild olive leaf extracts, whereas Chemlali leaf extracts contained only the first two. Analysis of olive pomace extracts revealed four major compounds: coniferyl alcohol, glyceryl monooleate, stigmast‐5‐en‐3‐ol, and methylursolate, present in both varieties, with significant variations in their peak areas. Cytotoxic evaluation showed that olive waste extracts significantly inhibited cancer cell growth and viability in both prostate (PC3) and breast (MDA‐MB‐231) cancer cell lines. Specifically, pomace extracts exhibited the strongest effect against PC3 prostate cancer cells, while leaf extracts were more effective against MDA‐MB‐231 breast cancer cells. These extracts inhibited cell proliferation, induced morphological and phenotypic alterations, modified the cell cycle progression, promoted apoptotic nuclear changes, and triggered apoptosis. Notably, wild olive extracts demonstrated stronger cytotoxic effects than those derived from the Chemlali cultivar. These findings highlight olive by‐products as promising sources of natural anticancer agents for pharmaceutical applications.

## 1. Introduction

Cancer remains one of the leading causes of death worldwide and a major barrier to increasing life expectancy. In 2020, approximately 19.3 million new cases were diagnosed, resulting in 10 million deaths globally [1]. Among the various types of cancer, lung cancer is the most frequently diagnosed, in both men and women. Its global incidence is projected to increase by 66.7%, with mortality estimated to reach 59.8% by 2040. Prostate cancer, the second most common cancer after lung cancer, recorded 1.4 million new cases and 375,304 deaths in 2020, accounting for 3.8% of cancer‐related deaths [2–4]. By 2040, an estimated 2.29 million new cases are expected.

Breast cancer remains the leading cause of cancer‐related deaths among women, with around 685,000 deaths and 2.3 million new cases reported in 2020. Projections suggest that the burden of this disease could exceed 3 million new cases by 2040 [5]. While chemotherapy and radiotherapy are common conventional treatments, they are often associated with significant limitations, including severe side effects and the development of drug resistance. As a result, medicinal plants with secondary metabolites exhibiting promising anticancer properties are gaining attention as potential therapeutic alternatives. These plants offer a valuable research opportunity for the development of more effective and less toxic cancer treatments.

The olive tree (*Olea europaea* L.), a member of the Oleaceae family, is one of the most widely cultivated fruit trees in the Mediterranean region, holding immense cultural, social, economic, and ecological significance [6]. However, olive oil production generates large amounts of solid and liquid waste, including leaves, pomace, and wastewater, which pose significant environmental challenges [7]. The valorization of these by‐products offers a promising solution, simultaneously generating economic benefits, supporting sustainable resource management, and minimizing environmental impact. Additionally, olive‐derived residues have been repurposed due to their high potential as a source of bioactive molecules [8].

Olive tree leaves are known for their health benefits, including antioxidant, anticancer [9, 10], anti‐inflammatory [11], antibacterial [12], antiviral [13], and antimicrobial properties [14, 15]. Furthermore, olive pomace can be utilized as an active ingredient in food or as a base for drug development, offering technological benefits such as antioxidant [16] and antibacterial properties [17]. However, its anticancer activity remains largely unexplored.

This study is aimed at analyzing the chemical composition of the hexane (HX) leaf fractions and the dichloromethane (DCM) pomace fractions from both cultivated Chemlali (var. *europaea*) and wild olive (var. *sylvestris*) varieties using GC‐MS analysis. Their anticancer effects were assessed in vitro by evaluating cell viability, cell cycle progression, and apoptosis induction in human prostate (PC3) and breast (MDA‐MB‐231) cancer cell lines treated with these extracts.

## 2. Material and Methods

### 2.1. Chemicals and Reagents

The solvents and reagents used in this study, including HX, DCM, ethanol (EtOH), and dimethyl sulfoxide (DMSO), were obtained from Scharlau (Spain). Culture media and supplements, such as Dulbecco′s Modified Eagle Medium (DMEM), fetal bovine serum (FBS), L‐glutamine, penicillin, and streptomycin, were all obtained from EuroClone (Milano, Italy). The 3‐(4,5‐dimethylthiazol‐2‐yl)‐2,5‐diphenyltetrazolium bromide (MTT) reagent was purchased from Sigma‐Aldrich (St. Louis, Missouri), and the Annexin V‐FITC detection kit was obtained from Miltenyi Biotec (Bologna, Italy). Additional reagents included propidium iodide (PI)/RNase staining solution and Hoechst 33258, which were purchased from Sigma‐Aldrich (St. Louis, Missouri). Etoposide was kindly provided by Dr. L. Zannini (CNR, Pavia).

### 2.2. Plant Materials

The plant material used in this study consists of leaves and olive pomace derived from two Tunisian olive cultivars: the Chemlali variety and the wild olive (*Oleaster*). The Chemlali variety originates from the Matmata region in southeastern Tunisia, known for its arid climate, and the wild olive tree is located in Ichkeul Park in northern Tunisia, a region characterized by a semiarid climate. Both cultivars were grown under natural conditions, without the application of fertilizers or pesticides. The olives and leaves of these two varieties were hand‐picked during the 2021–2022 harvest season. After collection, leaves were rinsed with distilled water, lyophilized, and ground into a fine powder. Fresh olive fruits were washed, defoliated, and crushed using a hammer mill (MM‐100, Type mc2, Sevilla, Spain). The resulting olive paste was mixed and then centrifuged using a two‐phase extraction method as previously described [17]. This process yielded olive oil and a by‐product known as pomace, consisting of fragments of olive skin, pulp, and pits. The pomace was dried at 50°C until a constant weight was achieved and then finely ground using a mechanical grinder (MM400 Mixer Mill, Retsch, Haan, Germany).

### 2.3. Preparation of Extracts

For extract preparation, 5 g of the leaf and pomace powders were macerated in 50 mL of 75% EtOH under overnight shaking. The filtrates collected were evaporated to dryness under reduced pressure using a rotary evaporator (Büchi R‐200, Switzerland) at 40°C. The EtOH crude extract was then resuspended in deionized water and fractionated using two organic solvents of increasing polarity: HX and DCM. Each fraction was concentrated under reduced pressure. The resulting sediments were dissolved in 75% EtOH and stored at −20°C until further analysis. This fractionation procedure was adapted from a previously established method [18]. The extraction yield (*Y*) for each fraction was determined using the following equation:

Y%=mM×100,

where *m* is the mass of the dried extract residue (g) and *M* is the initial weight of the powdered plant material (g). The extraction and fractionation processes were performed in triplicate on independent samples to ensure reproducibility and consistency.

Hence, four extracts were utilized in this study: HX fraction of Chemlali leaves (CL), HX fraction of wild *Oleaster* leaves (WL), DCM fraction of Chemlali pomace (CP), and DCM fraction of wild *Oleaster* pomace (WP).

### 2.4. GC‐MS Analysis

The chemical composition of the HX fraction of leaves and the DCM fraction of pomace from the two olive cultivars was analyzed through gas chromatography using an Agilent 7890 A interfaced with a 5975C mass spectrometer (5975C inert XL MSD, Agilent Technologies, Palo Alto, California, United States) employing electron impact ionization of 70 eV. A single microliter of each sample solution was introduced into the GC system equipped with a capillary column (Agilent 19091S‐433, HP‐5MS 5% Phenyl Methyl Silox, 30 m × 250 *μ*m × 0.25 *μ*m). The column temperature was programmed to increase from 50°C to 240°C at a rate of 5°C/min. Helium served as the carrier gas at a flow rate of 1.2 mL/min, and the split ratio was 10:1. The mass range covered 50–1000 *m*/*z*. The identification of the active compound involved comparing the retention time and recorded mass spectra with those stored in the Wiley/NBS mass spectral library (Wiley 9th/NIST 2017).

### 2.5. Cell Lines and Cell Culture

The breast cancer cell line MDA‐MB‐231 (American Type Culture Collection, RRID: CVCL_0062) was cultured in DMEM high glucose supplemented with 10% FBS, 1% L‐glutamine, and 1% penicillin–streptomycin [19]. The prostate cancer cell line PC3 (kind gift from H. De Jonge, RRID: CVCL_0035) was cultured in DMEM low glucose with 10% FBS, 1% L‐glutamine, and 1% penicillin–streptomycin, with the addition of 1% nonessential amino acids (NEAAs) [20]. Cell lines were carefully amplified and cryopreserved for use with no further authentication. All cell lines were mycoplasma‐free and maintained at 37°C in a humidified incubator with 5% CO_2_.

### 2.6. Cell Viability

Cell viability was assessed using the MTT colorimetric assay, following the method described by Maccario et al. [21]. Cells were seeded in 96‐well plates at a density of 1 × 10^4^ cells per well. The cells were exposed to different concentrations of the four fractions (1.95, 3.9, 7.81, 15.62, 31.25, 62.5, 125, 250, and 500 *μ*g/mL) for 48 h. For the control wells, one contained only medium, while the other included EtOH at a final concentration of 0.1% as a vehicle‐only control (CV). The 75% EtOH was used to dilute all extracts, and the 0.1% concentration was selected for its nontoxic effect on cells. Subsequently, the diluted extracts were discarded and replaced with 100 *μ*L/well of MTT solution (40 *μ*g/well in complete culture medium). After 3 h incubation, the MTT solution was substituted with 100 *μ*L/well of DMSO. After shaking, the absorbance (OD) was measured at 620 nm using a microplate reader (Multiskan FC, Thermo Fisher Scientific). Viability of cells exposed to treatments was calculated as follows: *%*Viability = (treated OD∗100)/untreated OD.

### 2.7. Analysis of Cell Morphology

Cells were seeded at a density of 15 × 10^5^ cells per dish and treated with CL and WL (100 and 200 *μ*g/mL) and CP and WP (200 and 300 *μ*g/mL), along with a solvent control consisting of EtOH (0.1%). After 48 h of treatment, morphological changes were assessed by optical microscopy.

### 2.8. Cell Cycle Analysis

The cell cycle profile was analyzed using PI staining, following the protocol adapted from Sassi et al. [22]. Cells were seeded in 25‐cm^2^ culture flasks at a concentration of 1 × 10^6^ cells per flask and incubated for 48 h with different treatment concentrations: 100 and 200 *μ*g/mL of the HX fraction from olive leaves (CL and WL), 200 and 300 *μ*g/mL of the DCM fraction from olive pomace (CP and WP), and a solvent control (EtOH 0.1%). After treatment, nonadherent and adherent cells, detached with trypsin, were combined and pelleted before fixation in 70% cold EtOH at −20°C for at least 1 h. Fixed cells were then washed with PBS and resuspended in PBS containing 5 *μ*g/mL PI, 2 mg/mL RNase A, and 0.05% NP‐40. Staining occurred for at least 30 min at room temperature (RT) and ON at 4°C. Ten thousand cells were measured for each sample with a BD FACS Lyric cytometer (CGS, University of Pavia) and analyzed with the Attune NxT software v February 4, 1627.1.

### 2.9. Observation of Nuclear Morphology Changes Using Hoechst 33258 Staining

PC3 and MDA‐MB‐231 cells were plated at a density of 1.5 × 10^5^ cells per dish and incubated for 48 h with CL and WL extracts (100 and 200 *μ*g/mL), CP and WP extracts (200 and 300 *μ*g/mL), or a solvent control (EtOH 0.1%). To identify apoptotic cells, Hoechst 33258 staining was performed. After fixation in cold 70% EtOH and washing with PBS, cells were stained with 0.5 *μ*g/mL Hoechst 33258 for 10 min at RT in the dark. Apoptotic cells were then visualized using fluorescence microscopy (Nikon Eclipse E400).

### 2.10. Annexin V/PI Assay

Flow cytometric analysis using the Annexin V‐FITC/PI assay (Miltenyi Biotec, Germany) was performed according to the manufacturer′s instructions to evaluate the type of cell death by measuring the proportions of viable, early apoptotic, late apoptotic, and necrotic cells following treatment with extracts. Briefly, cells were seeded in 25‐cm^2^ culture flasks at a density of 1 × 10^6^ and incubated for 48 h with IC_50_ concentrations for each extract, alongside etoposide (25 *μ*M for 16 h) as a proapoptotic control. After treatment, nonadherent and adherent cells harvested with trypsin were combined, washed with buffer, and resuspended in Annexin V binding buffer. Subsequently, 5 *μ*L of Annexin V‐FITC was added, and the mixture was incubated in the dark at RT for 15 min. The cells were then centrifuged, resuspended in 500 *μ*L of Annexin V binding buffer, and counterstained with 5 *μ*L of PI [23]. Finally, cell populations were measured using a BD FACS Lyric cytometer (CGS, University of Pavia) and analyzed with the Attune NxT software v February 4, 1627.1.

## 3. Results

### 3.1. Extract Analysis

**Figure 1:**
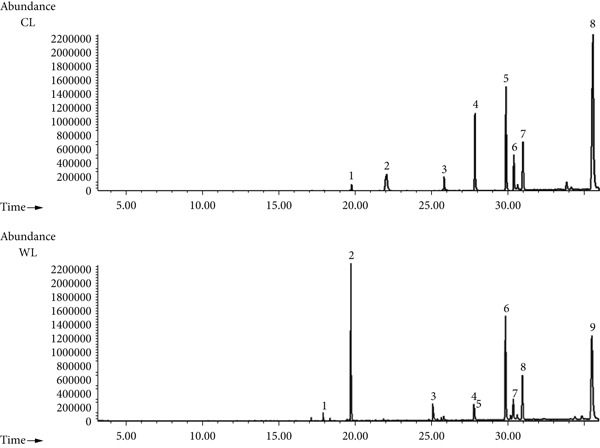
GC‐MS chromatogram of the HX fraction of the Chemlali leaf (CL) and wild olive leaf (WL) cultivars.

The extraction yields, expressed as percentages (%), varied significantly ( ^∗^ *p* < 0.05) among the different olive extracts (Figure S1). Leaf extracts yielded higher amounts of extractable material than pomace extracts, with WL exhibiting the highest extraction yield (1.88%), followed by CL (1.72%), while WP and CP showed significantly lower yields, not exceeding 0.80%. The GC‐MS analysis of leaf fractions identified eight compounds in CL (Figure 1, Table 1) and nine compounds in WL (Figure 1, Table 2). In CL, two major peaks were observed, corresponding to stigmast‐5‐en‐3‐ol (C_29_H_50_O) and erythrodiol (C_30_H_50_O_2_), with peak areas of 12.29% and 37.56%, respectively. In WL, three dominant compounds were detected: phytol (C_20_H_40_O, 19.69%), stigmast‐5‐en‐3‐ol (21.14%), and erythrodiol (33.69%).

**Table 1:** Chemical composition of the HX fraction of the Chemlali olive leaf (CL) cultivar.

**Peak**	**Retention time (min)**	**Area (%)**	**Compound name**	**Chemical formula**
1	19.787	0.74	Phytol	C_20_H_40_O
2	22.071	6.42	Uvaol	C_30_H_50_O_2_
3	25.837	1.60	Alpha‐Tocospiro B	C_28_H_48_O_4_
4	27.861	8.42	Alpha‐tocopherol quinone	C_26_H_44_O_3_
**5**	**29.893**	**12.29**	**Stigmast-5-en-3-ol**	C_29_H_50_O
6	30.401	5.48	*β*‐Amyrin	C_30_H_50_O
7	31.002	8.14	Alpha‐amyrin	C_30_H_50_O
**8**	**35.584**	**37.56**	**Erythrodiol**	C_30_H_50_O_2_

*Note:* The major compounds are shown in boldface texts.

**Table 2:** Chemical composition of the HX fraction of the wild olive leaf (WL) cultivar.

**Peak**	**Retention time (min)**	**Area (%)**	**Compound name**	**Chemical formula**
1	17.949	1.01	2‐Pentadecanone,6,10,14‐trimethyl	C_18_H_36_O
**2**	**19.769**	**19.69**	**Phytol**	C_20_H_40_O
3	25.135	3.93	13‐Docosenamide	C_22_H_45_NO
4	27.820	1.91	Vitamin E	C_29_H_50_O_2_
5	27.864	2.18	Alpha‐tocopherol quinone	C_26_H_44_O_3_
**6**	**29.894**	**21.14**	**Stigmast-5-en-3-ol**	C_29_H_48_O
7	30.400	4.82	*β*‐Amyrin	C_30_H_50_O
8	31.003	10.47	Alpha‐amyrin	C_30_H_50_O
**9**	**35.550**	**33.69**	**Erythrodiol**	C_30_H_50_O_2_

*Note:* The major compounds are shown in boldface texts.

**Figure 2:**
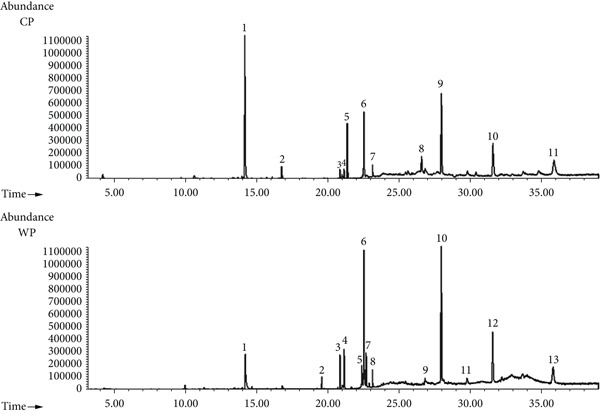
GC‐MS chromatogram of the DCM fraction of Chemlali pomace (CP) and wild olive pomace (WP) cultivars.

For the pomace fraction CP and WP, 11 and 13 compounds were identified, respectively (Figure 2, Tables 3 and 4). The four major compounds were coniferyl alcohol (C_10_H_12_O_3_), glyceryl monooleate (C_21_H_40_O_4_), stigmast‐5‐en‐3‐ol (C_29_H_50_O), and methylursolate (C_31_H_50_O_3_). The peak areas for CP were 28.59%, 10.30%, 16.58%, and 10.60%, while the peak areas for WP were 7.89%, 19.88%, 23.01%, and 11.62%, respectively.

**Table 3:** Chemical composition of the DCM fraction of Chemlali olive pomace (CP).

**Peak**	**Retention time (min)**	**Area (%)**	**Compound name**	**Chemical formula**
1	**14.178**	**28.59**	**Coniferyl alcohol**	**C**_**10**_ **H**_**12**_ **O**_**3**_
2	16.746	1.99	Trans‐sinapyl alcohol	C_11_H_14_O_4_
3	20.844	0.88	6‐Methylindole	C_9_H_9_N
4	21.130	1.25	2‐Monopalmitin	C_19_H_38_O_4_
5	21.347	7.58	Ethyl *N*‐phenylalaninate	C_11_H_15_NO_2_
6	**22.520**	**10.30**	**Glyceryl monooleate**	**C**_**21**_ **H**_**42**_ **O**_**4**_
7	23.131	1.08	13‐Docosenamide	C_22_H_43_NO
8	26.562	3.53	Gamma‐sitosterol	C_29_H_50_O
9	**27.950**	**16.58**	**Stigmast-5-en-3-ol**	**C**_**29**_ **H**_**48**_ **O**
10	**31.574**	**10.60**	**Methylursolate**	**C**_**31**_ **H**_**48**_ **O**_**3**_
11	35.859	6.54	Olean‐12‐en‐28‐oic acid	C_30_H_48_O_3_

*Note:* The major compounds are shown in boldface texts.

**Table 4:** Chemical composition of the DCM fraction of wild olive cultivar pomace (WP).

**Peak**	**Retention time (min)**	**Area (%)**	**Compound name**	**Chemical formula**
1	**14.192**	**7.89**	**Coniferyl alcohol**	**C**_**10**_ **H**_**12**_ **O**_**3**_
2	19.565	1.06	2‐Pyrrolidinone, 1‐(9‐octadecenyl)	C_22_H_41_NO
3	20.846	2.66	6‐Methylindole	C_9_H_9_N
4	21.133	4.30	2‐Monopalmitin	C_19_H_38_O_4_
5	22.384	3.42	1H‐Indole, 3‐methyl	C_9_H_9_N
6	**22.523**	**19.88**	**Glyceryl monooleate**	**C**_**21**_ **H**_**42**_ **O**_**4**_
7	22.685	3.93	Octadecanoic acid, 2,3‐dihydroxypropyl ester	C_21_H_42_O_4_
8	23.130	1.33	13‐Docosenamide	C_22_H_43_NO
9	26.813	1.13	3‐Ethoxy‐4‐methoxybenzaldehyde	C_10_H_12_O_3_
10	**27.945**	**23.01**	**Stigmast-5-en-3-ol**	**C**_**29**_ **H**_**48**_ **O**
11	29.753	1.59	9,19‐Cyclolanost‐25‐en‐3‐ol, 24‐methyl‐, (3*β*.,24S)	C_31_H_52_O
12	**31.560**	**11.62**	**Methylursolate**	**C**_**31**_ **H**_**48**_ **O**_**3**_
13	35.799	6.75	Olean‐12‐en‐28‐oic acid	C_30_H_48_O_3_

*Note:* The major compounds are shown in boldface texts.

### 3.2. Olive Extracts Reduce Cancer Cell Growth and Viability

**Figure 3:**
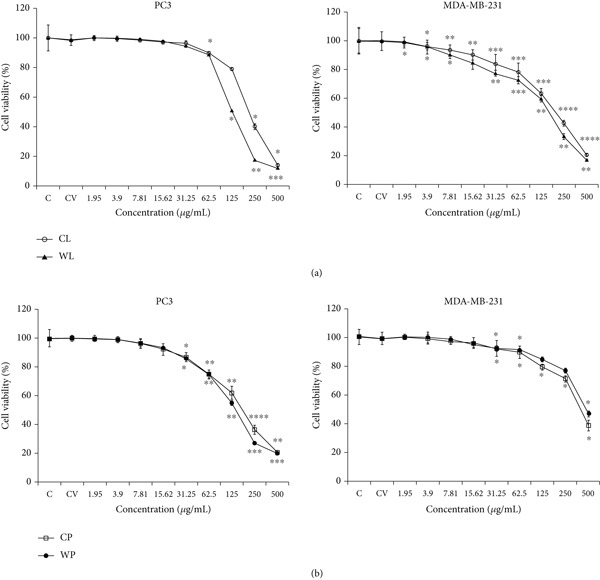
Dose‐dependent effect of olive extracts on the viability of PC3 and MDA‐MB‐231 cancer cell lines. Cells were treated for 48 h with increasing concentrations of (a) leaf and (b) pomace extracts and assessed using the MTT colorimetric assay. Data presented as mean ± SD of two independent experiments. Data were expressed as a percentage relative to the vehicle‐only control cells.  ^∗^ *p* < 0.05,  ^∗∗^ *p* < 0.01,  ^∗∗∗^ *p* < 0.001, and  ^∗∗∗∗^ *p* < 0.0001.

The cytotoxic and antiproliferative effects of olive extracts were assessed on PC3 and MDA‐MB‐231 cancer cell lines treated with varying concentrations of leaf (CL and WL) or pomace (CP and WP) extracts for 48 h (Figure 3, Table 5). A concentration‐dependent reduction in cell viability was observed with all extracts. For PC3, a reduction in cell viability was observed after CL and WL extract treatment, with significance starting from 62.5 *μ*g/mL, in which a reduction of about 12% was observed in comparison to the vehicle‐treated cells (*p* < 0.05). Increasing the concentration of the WL extract induced a reduction of cell viability that was stronger in comparison to the CL extract, albeit reaching comparable cell viability (about 10%) at the highest concentration of treatment (500 *μ*g/mL). The IC_50_ values were 217.58 *μ*g/mL for CL and 134.7 *μ*g/mL for WL. For the CP and WP extracts, a significant reduction in viability was first seen at 15.62 *μ*g/mL, with a decrease of 10% in viable cells (*p* < 0.05). Increasing the concentration of treatment up to 500 *μ*g/mL for either extract showed a similar reduction of viable cells, with high statistical significance in comparison to the vehicle cells. The IC_50_ values were 182.82 *μ*g/mL for CP and 145.74 *μ*g/mL for WP. For MDA‐MB‐231 cells, olive pomaces showed notably lower cytotoxicity than leaf extracts. In particular, CL and WL started to be cytotoxic in a significant manner at 3.9 *μ*g/mL, reaching high toxicity at 500 *μ*g/mL with a reduction of 80%. The IC_50_ values were 206.4 *μ*g/mL (CL) and 182.45 *μ*g/mL (WL) for the leaf products versus notably higher values of 427.17 *μ*g/mL (CP) and 441.92 *μ*g/mL (WP) for the pomaces.

**Table 5:** The 50% inhibitory concentration (IC_50_) values of olive extracts on PC3 and MDA‐MB‐231 cell lines.

**Cell lines**	**Olive extracts**			
	**CL**	**WL**	**CP**	**WP**
PC3	217.58 ± 23.9	134.7 ± 22.63	182.82 ± 12.06	145.74 ± 14.39
MDA‐MB‐231	206.4 ± 30.12	182.45 ± 26.36	427.17 ± 54.76	441.92 ± 82.13

Cells were treated with various concentrations of CL, WL, CP, and WP extracts for 48 h assessed using the MTT colorimetric assay. Cytotoxicity was assessed by measuring absorbance at 620 nm using a plate reader. The IC_50_ values are expressed in *μ*g/mL ± SD as the mean of three independent experiments. Based on the IC_50_ values obtained for PC3 cells, no significant difference was observed between the olive leaf and pomace extracts. Therefore, we chose to continue the analyses with the pomace extract, as it has been less extensively studied. In the case of the MDA‐MB‐231 breast cancer cell line, the leaf extract was selected for subsequent experiments as it proved to be the most effective. Doses observed to significantly decrease the viability were selected (i.e., CP and WP extracts were used at 200–300 *μ*g/mL and CL and WL extracts at 100–200 *μ*g/mL).

### 3.3. Cells Treated With Olive Extracts Show Altered Cell Morphology

Changes in cell morphology for PC3 and MDA‐MB‐231 cells treated for 48 h with olive extracts were monitored by microscopy (Figure 4). For PC3 cells treated with CP and WP at 200 and 300 *μ*g/mL, a reduction of cell density was evident, and irregular aggregates were visible with an altered cell morphology (Figure 4a). For MDA‐MB‐231 cells, exposure to olive extracts (CL and WL) caused reduced cell density, with visible cell condensation and shrinkage compared to the control and vehicle‐only samples (Figure 4b). The reduction in cell density appeared more pronounced at higher extract concentration, where a fibroblastic morphology was observed. Alterations in cell phenotype were confirmed in both treated cell lines by flow cytometry readings (Figure S2), which indicated a dose‐dependent shift in terms of cell size (forward scatter) and even more clearly in cell granularity (side scatter) in cells treated with olive extracts compared to CVs (Figure 4c,d).

Figure 4Changes in cell density and morphology in (a) PC3 and (b) MDA‐MB‐231 cancer cell lines upon 48 h of exposure to olive extract. Scale bar: 20 *μ*m. Effect of olive extracts on cell granularity (SSC) in (c) PC3 and (d) MDA‐MB‐231 cells. Representative dataset from two independent biological replicates.  ^∗∗∗^ *p* < 0.001 and  ^∗∗∗∗^ *p* < 0.0001.

**Figure:**
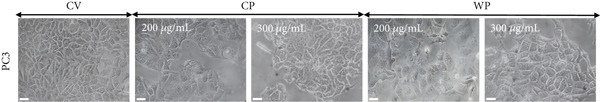
(a)

**Figure:**
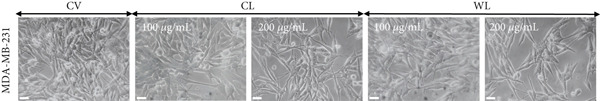
(b)

**Figure:**
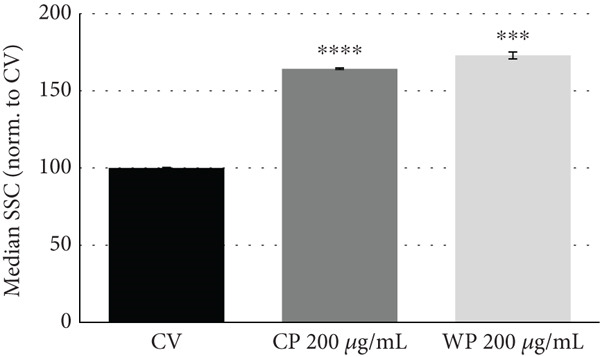
(c)

**Figure:**
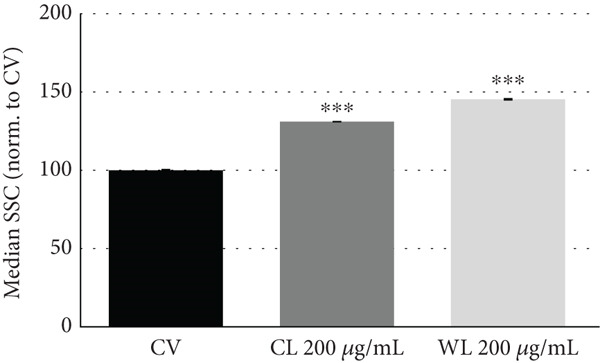
(d)

### 3.4. Olive Extracts Affect the Cell Cycle of PC3 and MDA‐MB‐231 Cells

**Figure 5:**
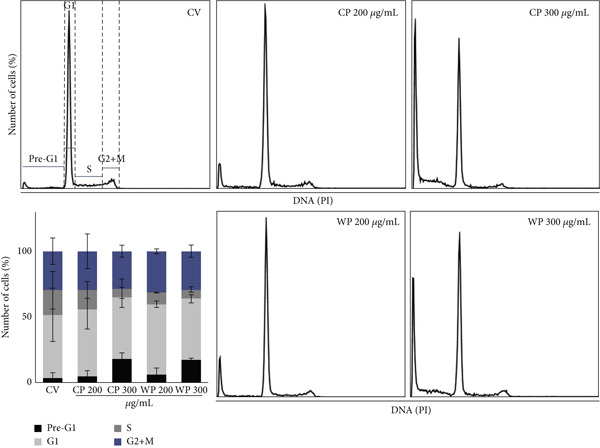
Effects of olive extracts on the cell cycle profile of PC3 cells after 48 h. Percentage of cells in each phase of the cell cycle for PC3 cells treated with CP and WP (200 and 300 *μ*g/mL), compared to the vehicle‐only control (CV), analyzed in two independent biological repeats.

To further analyze the effects of olive extracts on the two cancer models, the distribution of control and treated cells across the different phases of the cell cycle was measured using flow cytometry. Representative results of the cell cycle distribution are shown in Figure 5 with the corresponding quantitative analysis. Treatment of PC3 cells with 200 and 300 *μ*g/mL of CP and WP resulted in an increase in the sub‐G1 phase with a concomitant reduction in the S phase in comparison to vehicle control cells, particularly at the highest concentration. The sub‐G1 population, representing apoptotic cells and apoptotic bodies, was twice as high after treatment with 200 *μ*g/mL WP and 5.5 times higher after treatment with 300 *μ*g/mL WP or CP compared with the vehicle control group.

Exposure of WP and CP to a concentration of 200 *μ*g/mL increased the percentage of cells in the G1 phase by 53% and 50%, respectively, which was higher than the vehicle control (46%). Similarly, treatment with WP and CP at a concentration of 300 *μ*g/mL resulted in the G1 phase percentages of 45% and 47%, respectively. In addition, treatment of WP and CP with a concentration of 200 *μ*g/mL resulted in a more evident reduction in the proportion of cells in the S phase by 8% and 15%, respectively, while the value for the vehicle control sample was 19%. At a concentration of 300 *μ*g/mL, only 6% of cells were in the S phase for both extracts. However, no differences were observed in the number of cells in the G2/M phase in treated PC3 cells in comparison to the vehicle control group.

**Figure 6:**
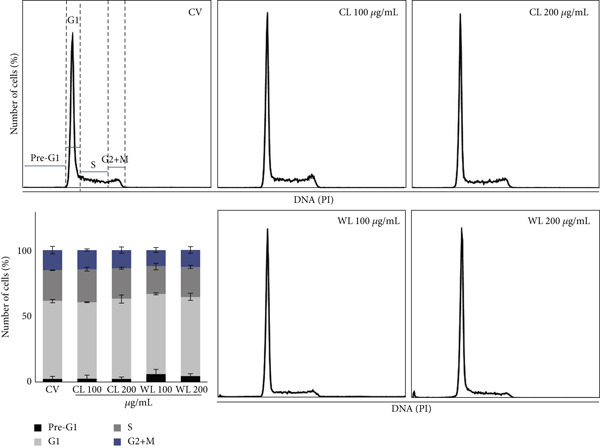
Effects of olive extracts on the cell cycle profile of cells after 48 h. Percentage of cells in each phase of the cell cycle for MDA‐MB‐231 cells treated with CL and WL (100 and 200 *μ*g/mL), compared to the vehicle‐only control (CV), analyzed in two independent biological repeats.

In MDA‐MB‐231 (Figure 6), 48 h of treatment with 100 and 200 *μ*g/mL of CL and WL induced minor variations in the G1, S, and G2/M phases, with a mild accumulation of cells in the pre‐G1 phase, in particular after treatment with WL, in comparison to the vehicle control group. After 100 *μ*g/mL WL treatment, the sub‐G1 population was 8.56% in comparison to the vehicle control group (3.6%). This increase was accompanied by a stable accumulation of cells in the G1 phase (61% vs. 59.9% in vehicle control cells) and a reduction in the S phase (19% vs. 23% in vehicle control cells). With a higher treatment concentration (200 *μ*g/mL), no further modification was observed.

In these samples, nuclear morphology observed by fluorescence microscopy using Hoechst 33258 (Figure 7) confirmed the presence of nuclei with apoptotic characteristics, including nuclear shrinkage, chromatin condensation, and nuclear fragmentation, visible in PC3 samples (Figure 7a). Similar results, albeit less evident, were observed in MDA‐MB‐231 after treatment with olive extracts (CL and WL) (Figure 7b).

Figure 7Nuclear staining of cells treated for 48 h with olive extracts. (a) PC3 cells treated with CP and WP (200 and 300 *μ*g/mL). (b) MDA‐MB 231 cells treated with CL and WL (100 and 200 *μ*g/mL). Scale bar: 20 *μ*m.

**Figure:**
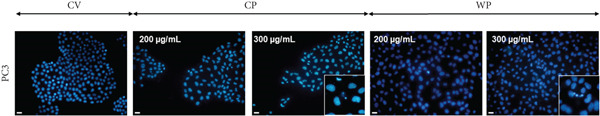
(a)

**Figure:**
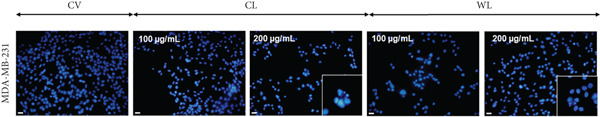
(b)

### 3.5. Treatment With Olive Extracts Increases the Apoptotic Fraction in Cancer Cell Lines

The increase in the pre‐G1 phase observed upon treatment with the olive extracts was further explored by Annexin V and PI staining to assess the apoptotic fractions in each line (Figure 8). Flow cytometric measurements of PC3 treated for 48 h with pomace (CP and WP) olive extracts showed an increase in the late apoptotic population compared to CVs, which was particularly visible with WP (Figure 8a), which increased late apoptotic events (27.4%) in comparison to the control cells (21%), while for the etoposide‐treated cells used as a positive control, a percentage of 36.8% was observed.

Figure 8Effects of olive extracts on the apoptotic fraction in PC3 and MDA‐MB‐231 cells. (a) PC3 and (b) MDA‐MB‐231 cells were treated for 48 h with CP and WP (200 and 300 *μ*g/mL) and CL and WL (100 and 200 *μ*g/mL), respectively. Representative flow cytometry dot‐blots (left panels) showing Annexin V (in *x*) and PI (in *y*) with corresponding quantification of the early and late apoptotic fractions in each line (right panels). The data represent the changes in percentage of cells in early/late apoptotic phases, compared to corresponding controls. Data from two independent experiments.

**Figure:**
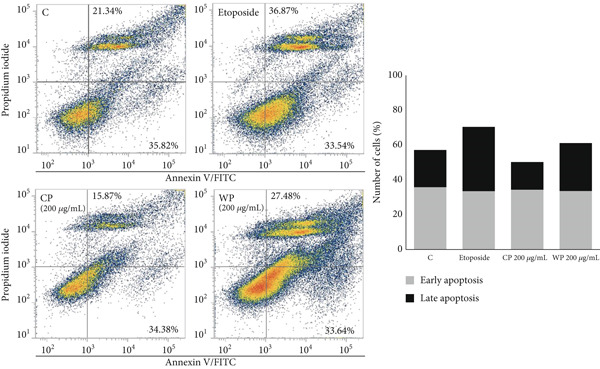
(a)

**Figure:**
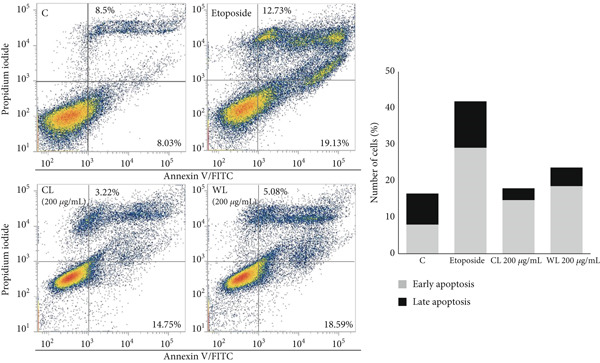
(b)

In accordance with the previous observations, MDA‐MB‐231 cells showed a different response after treatment with olive extracts (Figure 8b), with a more evident increase in the early apoptotic events when cells were treated with CL and WL at 200 *μ*g/mL for 48 h. The early apoptotic events increased from 8% in control cells to 14.7% and 18.5% for CL and WL, respectively. In contrast, the late apoptotic levels were higher in control cells (8.5%) in comparison to CL‐ and WL‐treated cells (3.2% and 5%, respectively). Etoposide‐treated cells showed high levels of both early and late apoptotic events (29% and 12.7%, respectively). The high level of early and late apoptotic events seen in PC3 cells was confirmed by the comet assay (data not shown), which showed similar levels of single‐strand breaks (SSBs) in control and pomace‐treated cells, while MDA‐MB‐231 cells showed levels of DNA damage approximately 40% lower in control cells.

## 4. Discussion

Medicinal plants have been widely recognized for their therapeutic potential, particularly due to their abundance of bioactive compounds with significant anticancer properties. These molecules exert their effects through various mechanisms, including inhibition of cell proliferation, induction of apoptosis, prevention of metastasis, and overcoming treatment resistance, making them promising alternatives or adjuncts to conventional therapies [24, 25]. *Olea europaea* L. is one such plant which has a special mention in medicinal science [26].

In this regard, our study explored the anticancer effects of olive leaves and pomace extracts from Chemlali (CL and CP) and wild olive (WL and WP) on cell growth, cell cycle distribution, and apoptotic induction in PC3 (prostate cancer) and MDA‐MB‐231 (breast cancer) cell lines. Preliminary biochemical analysis was performed to identify the extract composition. GC‐MS analysis of leaf extracts revealed that stigmast‐5‐en‐3‐ol and erythrodiol were the predominant compounds in both CL and WL extracts. In the CL extract, they accounted for 12.29% and 37.56% of the total composition, respectively, while in the WL extract, their proportions were 21.14% and 33.69%. Additionally, phytol was significantly more abundant in WL extract (19.69%) compared to CL extract (0.74%). These bioactive molecules are known for their anti‐inflammatory [27, 28], antioxidant [29], antidiabetic [30, 31], and antimicrobial [32] properties, and they also have well‐documented anticancer potential [33–35].

Similarly, GC‐MS analysis of olive pomace extracts revealed four key compounds, including stigmast‐5‐en‐3‐ol, which is also present in olive leaf extract, along with coniferyl alcohol, glyceryl monooleate, and methylursolate, identified in both varieties but exhibiting notable variations in peak areas. In the CP extract, these compounds were present at 16.58%, 28.59%, 10.30%, and 10.60%, respectively, while in the WP extract, their concentrations were 23.01%, 7.89%, 19.88%, and 11.62%. These molecules display diverse therapeutic properties, including antioxidant [36], vasodilatory [37, 38], anti‐inflammatory, anticancer, and antiparasitic effects [39]. Cytotoxicity assays demonstrated a decrease in cancer cell viability across all extracts, in a dose‐dependent manner. In PC3 cells, the WL extract exhibited greater potency than the CL extract, with IC_50_ values of 134.7 and 217.58 *μ*g/mL, respectively. This suggests that the higher phytol and stigmast‐5‐en‐3‐ol content in WL may contribute to its enhanced cytotoxic activity. Supporting this, Sakthivel et al. [40] reported that phytol exhibits potent antiproliferative effects on A549 cells in a dose‐ and time‐dependent manner, with IC_50_ values of 70.81 ± 0.32 * μ*M at 24 h and 60.7 ± 0.47 * μ*M at 48 h of treatment. Similarly, de Alencar et al. [41] demonstrated that phytol significantly reduced cell viability and the rate of division in sarcoma (S‐180) and human leukemia (HL‐60) cells, with IC_50_ values of 18.98 ± 3.79 and 1.17 ± 0.34 * μ*M, respectively. Additionally, stigmast‐5‐en‐3‐ol has been shown to exert antiproliferative effects against leukemia (HL‐60), breast cancer (MCF‐7) [33], and melanoma cells [42].

Similarly, the WP extract displayed stronger cytotoxicity than the CP extract, with IC_50_ values of 145.74 and 182.82 *μ*g/mL, respectively. To our knowledge, this is the first study investigating the antiproliferative activity of wild olive pomace, while the cytotoxic potential of cultivated olive pomace has been reported in some studies. For instance, Goldsmith et al. [43] reported that a methanolic extract of olive pomace derived from the Frantoio cultivar, at a concentration of 100 *μ*g/mL, effectively inhibited the growth of several cancer cell lines. The strongest effects were observed in MCF‐7 (57%) and A2780 (53%) cells, followed by U87 (49%), SJ‐G2 (43%), and MIA PaCa‐2 (42%) cells. Likewise, Ramos et al. [44] demonstrated that an aqueous olive pomace extract obtained from a Portuguese olive oil factory in Alvito (Beja, Portugal), containing 33.36–45.81 mg GAE/g DW of phenolic compounds, exhibited both antiproliferative and antioxidant effects on MDA‐MB‐231 breast cancer cells. More recently, Ferreira et al. [45] reported that olive pomace extract composed of a blend of Cobrançosa, Madural, Verdeal Transmontana, Cordovil, and Arbosana varieties from two‐phase olive mills in Portugal significantly reduced cell proliferation across multiple cancer types, including breast (MCF‐7), pancreatic (AsPC‐1), and colorectal (HT29 and Caco‐2) cancer cells. Data from the present study suggest pomace extracts from the wild variety may represent a valuable resource with a biological activity profile deserving further analysis.

In MDA‐MB‐231 cells, however, pomace extracts exhibited lower cytotoxicity compared to leaf extracts. The IC_50_ values for CP and WP extracts were 427.17 and 441.92 *μ*g/mL, respectively. Similarly, Quero et al. [46] reported that after 72 h of incubation, the ohmic–hydroethanolic (OH‐EtOH) pomace extract inhibited Caco‐2 cell proliferation with an IC_50_ of 692.32 *μ*g/mL, whereas leaf extracts demonstrated greater potency, with IC_50_ values of 206.4 *μ*g/mL (CL) and 182.45 *μ*g/mL (WL). Moreover, numerous studies have highlighted the potent, dose‐ and time‐dependent cytotoxic effects of olive leaf extracts against various cancers, including human melanoma [47], prostate [48], pancreatic [49], neuroblastoma [50], colon [51], and liver cancer [52]. Other natural products beyond olive by‐products have demonstrated antiproliferative effects in PC3 and MDA‐MB‐231 cells, for example, *Ceratonia siliqua* [53], green tea [54], *Heliotropium indicum*, *Vernonia amygdalina*, *Dissotis rotundifolia*, and *Launaea taraxacifolia* [55], which were observed to inhibit PC3 cell growth. Similarly, *Nigella sativa* [56], *Lantana camara* [57], and *Tabernaemontana catharinensis* [58] were reported to reduce MDA‐MB‐231 cell viability. As seen for other medicinal plants, the results obtained here highlight that olive extracts, particularly from the wild cultivar, are able to influence cancer cell proliferation.

To further explore the mechanisms behind the antiproliferative effects of olive extracts on PC3 and MDA‐MB‐231 cell lines, the most effective extracts were selected based on the dose–response results: olive pomace extract for PC3 (200–300 *μ*g/mL) and leaf extracts for MDA‐MB‐231 (100–200 *μ*g/mL). After 48 h of treatment, cells treated with these olive extracts displayed significant changes in cell density and morphology. Additionally, alterations in cell phenotype were observed, indicating a shift in cell size (forward scatter) and granularity (side scatter) in treated cells compared to CVs, which was more evident at higher concentrations.

To investigate the underlying mechanisms, cell cycle progression and apoptosis induction were assessed. Olive extracts modified the cell cycle distribution of both cell lines. Our data show that treatment of PC3 cells with 200–300 *μ*g/mL of CP and WP extracts led to a dose‐related increase in the sub‐G1 population, indicating apoptosis, accompanied by a decrease in the S phase. Similarly, Quero et al. [46] reported that OH‐EtOH extracts altered the cell cycle dynamics in Caco‐2 cells, inducing G1‐ and S‐phase arrest while reducing the G2 phase compared to untreated controls. Consistent with our findings, Fernando et al. [33] demonstrated that stigmast‐5‐en‐3‐ol, identified in our analysis as a major compound in pomace, promotes apoptotic body formation, increases the accumulation of sub‐G1 apoptotic cells, and induces DNA damage in HL‐60 and MCF‐7 cells. It was also found to modulate key apoptotic regulators by upregulating Bax, Caspase‐3, and PARP cleavage while downregulating Bcl‐xL, thereby activating the mitochondria‐mediated intrinsic apoptotic pathway [33].

Furthermore, treatment of MDA‐MB‐231 cells with 100 and 200 *μ*g/mL of CL and WL extracts resulted in slight changes in the G1, S, and G2/M phases, along with a moderate accumulation of cells in the pre‐G1 phase, particularly following WL treatment, compared to vehicle‐treated controls. Our results are in line with previous studies, such as Limam et al. [59], who reported a significant increase in the sub‐G1 population and a marked decrease in the G1 phase in U266 cells, after a 24‐h exposure to 100 *μ*g/mL of *Olea europaea* L. cv. Chetoui leaf and stem extracts. Additionally, Goldsmith et al. [49] showed that aqueous olive leaf (AOL) extract induced a significantly greater S‐phase arrest in the HT29 cell line compared to PC3 cells. Moreover, Itoh et al. [60] demonstrated that phytol, identified in the wild variety leaf extract, induced cell death through the promotion of S‐phase cell arrest by producing reactive oxygen species (ROS). This mechanism would complement the findings of Thakor et al. [35] that phytol induces apoptosis via activation of TRAIL, FAS, and TNF‐*α* death receptors, leading to Caspase‐9 and Caspase‐3 activation. More recently, Yu et al. [61] confirmed that phytol suppresses cancer cell proliferation, migration, and survival by inhibiting the PI3K/Akt signaling pathway. In agreement, Allouche et al. [62] demonstrated that treatment of MCF‐7 cells with 100 *μ*M erythrodiol, derived from olive skin, resulted in a marked reduction in the percentage of cells in the G0/G1 phase (33%) and a notable increase in the sub‐G1 phase population, which was 10 times higher than that in untreated control cells. Hoechst 33258 staining and Annexin V staining provided complementary evidence of apoptosis induction by olive extracts in PC3 and MDA‐MB‐231 cells. Nuclear staining confirmed key apoptotic features, including nuclear shrinkage, chromatin condensation, and fragmentation, which align with the Annexin V results, showing a significant increase in apoptotic cell populations. In PC3 cells, both CP and WP extracts enhanced late apoptosis, with WP showing a more pronounced effect (27.4% vs. 21% in control), which was consistent with the nuclear damage observed in Hoechst‐stained cells. This effect was comparable to the positive control, etoposide (36.8% late apoptosis). Unexpectedly, PC3 control cells showed high levels of early and late apoptotic events, an observation in line with a comet assay, which confirmed similar levels of SSBs in control and pomace‐treated cells (data not shown). This suggests that PC3 cells have greater genetic instability than MDA‐MB‐231 cells, whose levels of DNA damage were approximately 40% lower in control cells. Similarly, in MDA‐MB‐231 cells, CL and WL extracts predominantly promoted early apoptosis (14.7% and 18.5% vs. 8% in control), correlating with nuclear condensation and fragmentation observed via Hoechst staining. These results are consistent with previous studies on olive extract–induced apoptosis [45, 58]. These observations also underline the more marked biological properties of wild olive extract in comparison to the Chemlali cultivar, with an induction of apoptosis of around 60% for WP and 25% for WL. Additionally, Sakthivel et al. [40] demonstrated that phytol, detected in wild olive leaf extract, induces apoptosis in A549 cells while exhibiting lower toxicity in normal cells, suggesting its potential as a therapeutic agent against lung cancer. Promotion of both early and late apoptosis has been reported for other medicinal plants, in PC3 with *Achillea wilhelmsii* extract [63] and anthocyanins from *Vitis coignetiae* Pulliat fruits [64], for instance. Similarly, in MDA‐MB‐231 cells, apoptosis was triggered by ferulic acid isolated from the *n*‐butanol fraction of *Cycas thouarsii* leaves [65], as well as by extracts from *Ephedra* and *Forsythia suspensa* leaves [66, 67]. These observations further support the potential of natural products as possible modulators of cancer cell death.

Taken together, the present findings open the possibility that the cytotoxic and proapoptotic effects of olive extracts may be linked to the presence of bioactive compounds such as stigmast‐5‐en‐3‐ol and phytol, which have been reported to promote apoptosis via ROS generation, disruption of mitochondrial membrane potential, and inhibition of the PI3K/Akt survival pathway [60, 62] and which were more abundant in the wild leaf extract. Future molecular investigations seeking to characterize the pathways activated by these compounds will be useful to decipher the signaling cascades involved.

## 5. Conclusions

This study offers the first evidence of the antiproliferative activity of wild olive pomace, with a comparative analysis between cultivated and wild varieties. This represents a major step forward in the valorization of olive by‐products (leaves and pomace), emphasizing their potential as valuable sources of bioactive compounds for therapeutic applications, with the wild olive cultivar appearing as the most promising in comparison to the Chemlali in particular, considering its chemical composition and the biological effects observed. These in vitro findings lay a strong foundation for further research, particularly in evaluating the key compounds identified through GC‐MS analysis, investigating their mechanisms of action, and assessing their in vivo efficacy to advance toward clinical applications. Considering the different results on the two cancer cell lines, it may be useful to further explore the specificity of different olive extracts for different cancer models, as well as to evaluate the selectivity of their cytotoxic effect in cancer cells compared to normal, nontumorigenic cell populations. New perspectives could also include the production of extracts in the absence of organic solvent, for instance, a rapid innovative solid–liquid dynamic extraction technology (RSLDE), using the Naviglio extractor [68] to increase the production of green compounds, minimizing environmental impact.

## Conflicts of Interest

The authors declare no conflicts of interest.

## Funding

This study was funded by the Tunisian Ministry of Higher Education and Scientific Research and the Italian Ministry of University and Research (MUR) (through the PhD program in Translational & Precision Medicine and a grant to the Department of Molecular Medicine of the University of Pavia under the initiative Dipartimenti di Eccellenza [2023–2027]).

## Supporting information


**Supporting Information** Additional supporting information can be found online in the Supporting Information section. Figure S1: Extraction yields expressed as percentages (%) among the different olive extracts (*n* = 3,  ^∗^ *p* < 0.05). Figure S2: (A) Changes in forward scatter and (B) side scatter in PC3 and MDA‐MB‐231 cells treated with olive extracts compared to vehicle‐only controls.

## Data Availability

Data are available from the authors on reasonable request.
