# Supplementary figures and images for: Antitumor Effects of Chemlali and Wild Olive Tree Extracts: Role in Cell Proliferation and Apoptosis in Prostate (PC3) and Breast (MDA‐MB‐231) Cancer Cell Lines
Source: Biomed Res Int. 2025 Dec 11;2025:4911257. doi: 10.1155/bmri/4911257 (PMC12699138; doi:10.1155/bmri/4911257)

## Slide 1
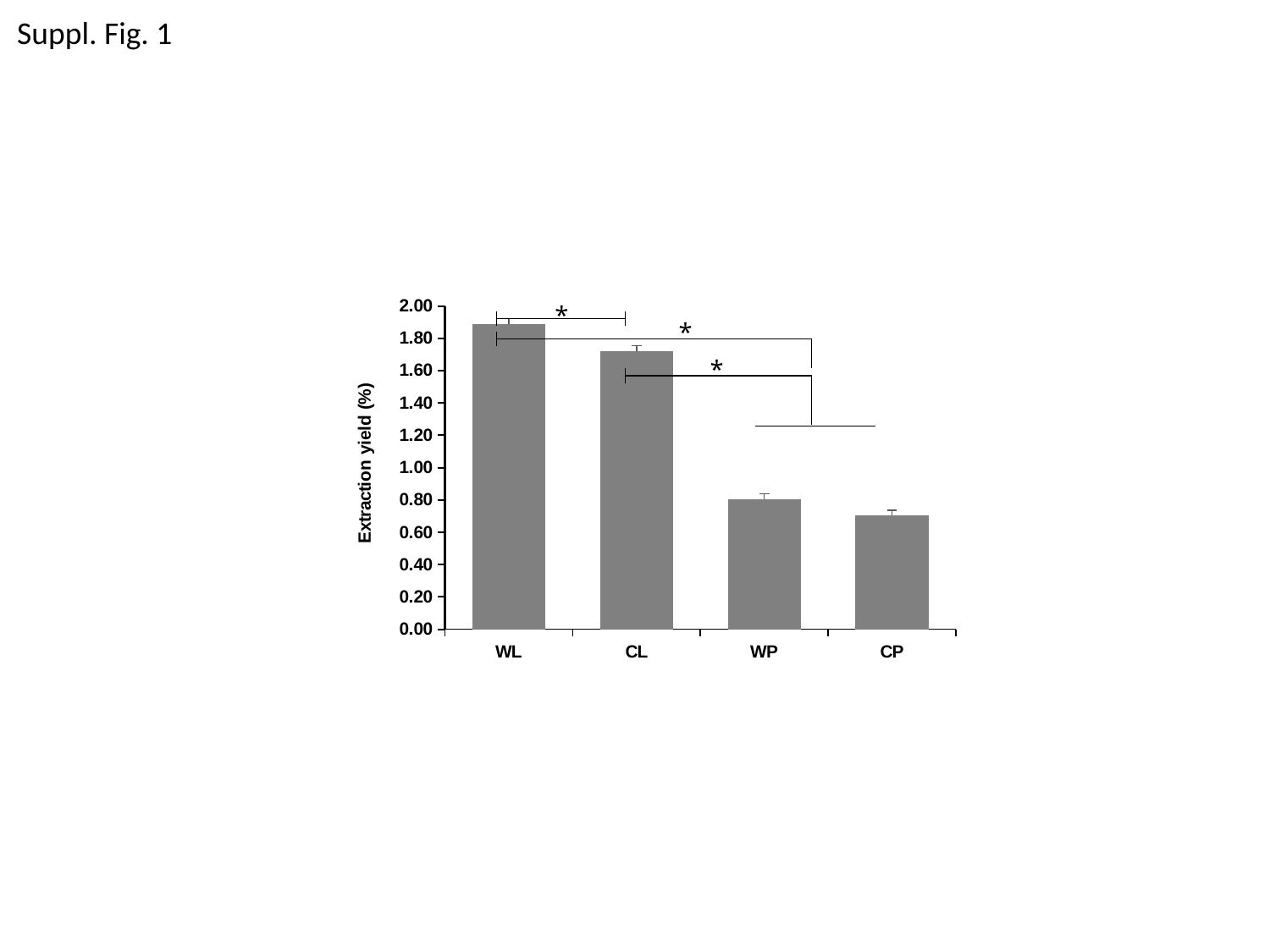

Suppl. Fig. 1
### Chart
| Category | Extraction yield (%) |
|---|---|
| WL | 1.88866666666667 |
| CL | 1.7210333333333834 |
| WP | 0.8053333333333348 |
| CP | 0.7036666666666723 |*
*
*

Supplement: Supplementary file 1 — Supporting Information Additional supporting information can be found online in the Supporting Information section. Figure S1: Extraction yields expressed as percentages (%) among the different olive extracts (n = 3, ∗ p < 0.05). Figure S2: (A) Changes in forward scatter and (B) side scatter in PC3 and MDA‐MB‐231 cells treated with olive extracts compared to vehicle‐only controls. [file BMRI-2025-4911257-s001.zip › Suppl Fig.1.pptx]

## Slide 1
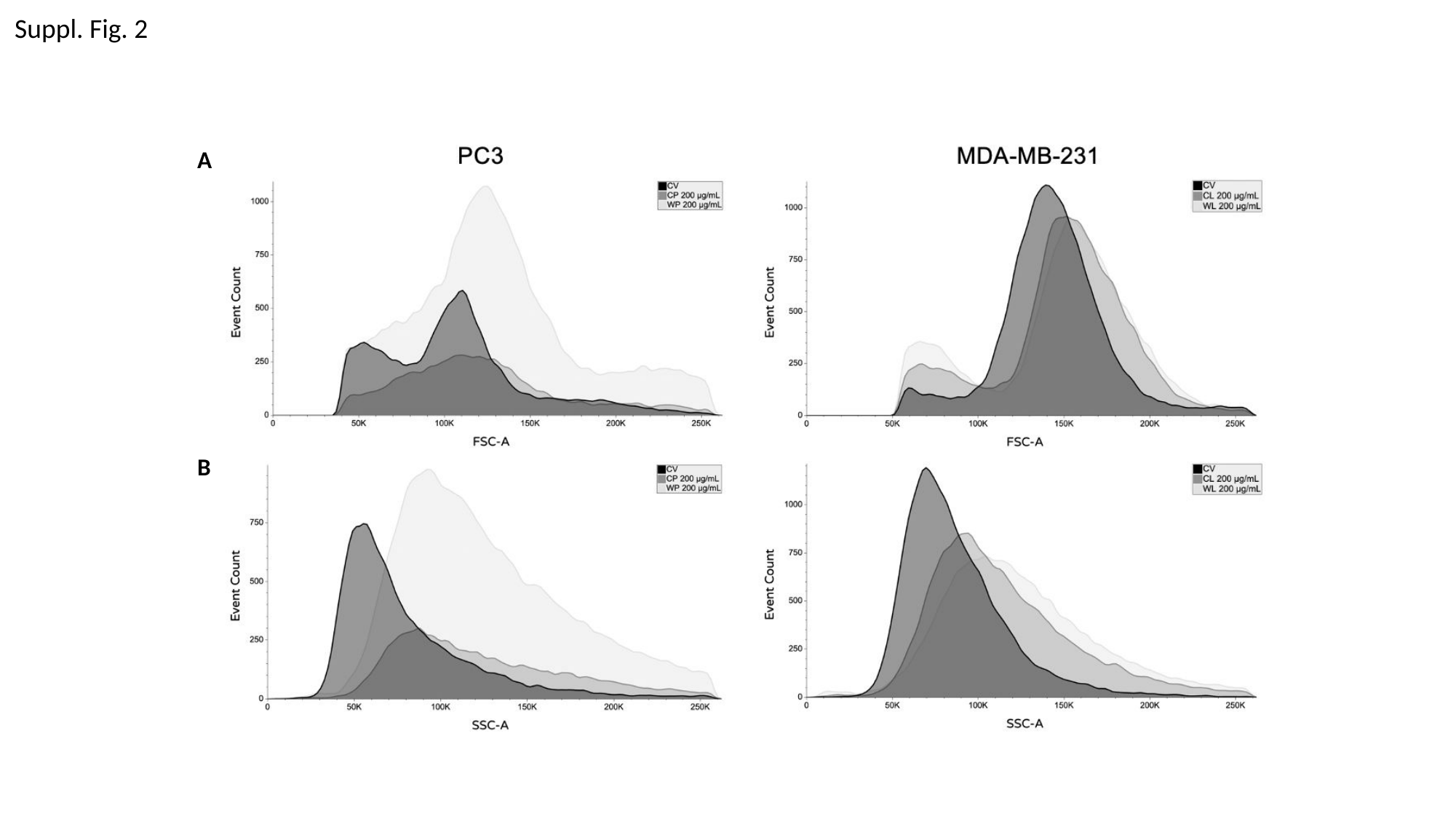

Suppl. Fig. 2
A
B

Supplement: Supplementary file 1 — Supporting Information Additional supporting information can be found online in the Supporting Information section. Figure S1: Extraction yields expressed as percentages (%) among the different olive extracts (n = 3, ∗ p < 0.05). Figure S2: (A) Changes in forward scatter and (B) side scatter in PC3 and MDA‐MB‐231 cells treated with olive extracts compared to vehicle‐only controls. [file BMRI-2025-4911257-s001.zip › Suppl Fig.2.pptx]
